# Importance of Maximal Strength and Muscle-Tendon Mechanics for Improving Force Steadiness in Persons with Parkinson’s Disease

**DOI:** 10.3390/brainsci10080471

**Published:** 2020-07-22

**Authors:** Rowan R. Smart, Cydney M. Richardson, Daryl J. Wile, Brian H. Dalton, Jennifer M. Jakobi

**Affiliations:** 1 School of Health and Exercise Sciences, University of British Columbia Okanagan, Kelowna, BC V1V1V7 Canada; rowan.smart@ubc.ca (R.R.S.); cydneyrichardson@hotmail.ca (C.M.R.); brian.dalton@ubc.ca (B.H.D.); 2 Okanagan Movement Disorders Clinic, Kelowna, BC V1Y1T2, Canada; dwile@mail.ubc.ca

**Keywords:** ultrasound, muscle fascicle, medial gastrocnemius, Achilles tendon

## Abstract

Although plantar flexion force steadiness (FS) is reduced in persons with Parkinson’s disease (PD), the underlying causes are unknown. The aim of this exploratory design study was to ascertain the influence of maximal voluntary contraction (MVC) force and gastrocnemius-Achilles muscle-tendon unit behaviour on FS in persons with PD. Nine persons with PD and nine age- and sex-matched non-PD controls (~70 years, 6 females per group) performed plantar flexion MVCs and sub-maximal tracking tasks at 5, 10, 25, 50 and 75% MVC. Achilles tendon elongation and medial gastrocnemius fascicle lengths were recorded via ultrasound during contraction. FS was quantified using the coefficient of variation (CV) of force. Contributions of MVC and tendon mechanics to FS were determined using multiple regression analyses. Persons with PD were 35% weaker during MVC (*p* = 0.04) and had 97% greater CV (*p* = 0.01) with 47% less fascicle shortening (*p* = 0.004) and 38% less tendon elongation (*p* = 0.002) than controls. Reduced strength was a direct contributor to lower FS in PD (ß = 0.631), and an indirect factor through limiting optimal muscle-tendon unit interaction. Interestingly, our findings indicate an uncoupling between fascicle shortening and tendon elongation in persons with PD. To better understand limitations in FS and muscle-tendon unit behavior, it is imperative to identify the origins of MVC decrements in persons with PD.

## 1. Introduction

Parkinson’s Disease (PD) is a chronic progressive neurological disorder that involves both motor and non-motor symptoms that commonly include bradykinesia, rigidity, tremor, and postural instability, which culminate in functional decline and disability [1]. The physiological changes underlying functional decline in PD have measurable correlates within the peripheral nervous system, muscle and muscle-tendon unit [2,3,4,5,6], thus providing an approach to understanding how force control contributes to functional limitations in persons with PD.

Force steadiness (FS), the ability to maintain a pre-established force at a target level, is used as a measure of functional performance [2,6,7], and in persons with PD, FS is less than in non-PD controls, independent of tremor [8,9,10,11]. FS varies with age, sex, muscle strength, and limb position, and is influenced by the nervous system and neuromuscular factors such as motor unit properties and tendon mechanics [12,13,14,15,16,17]. Plantar flexion strength and FS are less in persons with PD than healthy controls [8], but the reasons for these differences are unclear. In older adults—relative to young—the lower FS is associated with alterations in neural drive [14,18] and muscle-tendon mechanics [16]. During muscle contraction, a stiffer tendon elongates less per applied unit of force, which facilitates smooth transfer of force through a more rigid unit and enables a more consistent force output [15,16,17,19]. Evaluating contributions of muscle shortening and tendon mechanics to plantar flexion FS in persons with PD is important, as FS is a strong predictor of functional movement performance [2,6,7].

Mechanical properties of muscles and tendons in persons with PD have been described for the upper limb in a resting state using ultrasound elastography [20,21], and lower limb during passive ankle range of motion to evaluate gastrocnemius-Achilles tendon slack lengths [22]. However, mechanical properties of the gastrocnemius-Achilles tendon unit have not been quantified during active contraction in persons with PD. Quantifying tendon mechanics during active contractions across submaximal force levels is necessary to understand the interaction between muscle fascicle shortening and tendon elongation [23], as well as the load-dependent response across various submaximal forces [24,25]. Reductions in strength in persons with PD due to reduced voluntary activation [26] may decrease the active load placed on the tendon by the muscle, thereby reducing tendon elongation during contraction. It is unknown if muscle fascicle behavior is altered in persons with PD, and whether alterations in muscle-tendon unit interaction are a factor in reduced force control.

We conducted an exploratory study on the contribution of muscle-tendon mechanics to FS comparing persons with PD to age- and sex-matched healthy controls. We evaluated plantar flexion FS along with muscle strength, architectural and mechanical factors of the gastrocnemius-Achilles tendon unit in persons with PD compared to age- and sex-matched non-PD controls. We hypothesized that persons with PD would be weaker and have lower medial gastrocnemius muscle fascicle shortening during contractions, leading to less tendon elongation that would contribute to lower plantar flexion FS.

## 2. Materials and Methods

### 2.1. Ethical Approval

This study was approved by the University of British Columbia Clinical Research Ethics Board (no. H16-02963) and conformed to the standards set by the Declaration of Helsinki. All participants provided written informed consent prior to commencing the study.

### 2.2. Participants

Nine persons with PD, and 9 age- and sex-matched non-PD controls participated in this study (Table 1). Persons with PD were recruited from local Parkinson’s disease support groups. Age- and sex-matched non-PD controls were recruited from the local community. Seven of the PD participants were on prescribed dosages of levodopa/carbidopa for the management of symptoms, and all PD participants were tested during the ON phase of their medication cycle, ~1.5 h after medication intake. Participants were excluded if they had any neurological (unrelated to Parkinson’s disease) muscular, metabolic, or cardiovascular conditions that could affect force control, or lower-leg injuries in the 6 months prior to experimental testing. The motor examination portion of the Movement Disorders Society Unified Parkinson’s Disease Rating Scale (MDS-UPDRS) was conducted for each participant in the PD group.

### 2.3. Experimental Setup and Protocol

**Figure 1:**
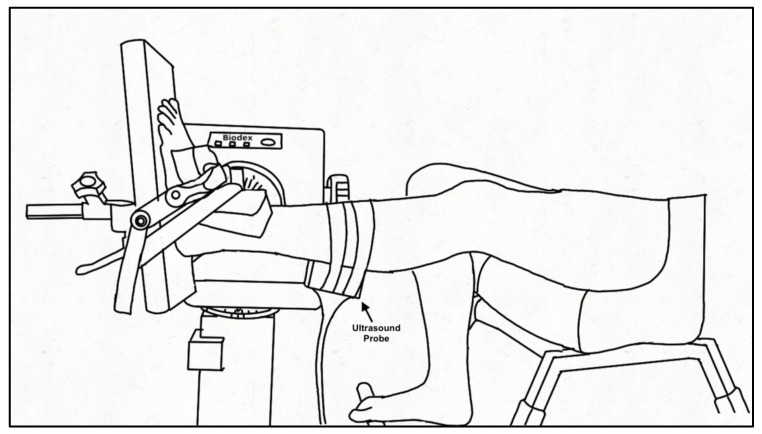
Experimental set-up. Schematic of participant seated in the Biodex dynamometer chair with the tested leg secured to the footplate of the dynamometer using inelastic straps. The ultrasound probe was encased in a customized probe holder and secured over the medial gastrocnemius using inelastic straps. The non-tested leg rested on a support.

Participants were seated in a Biodex dynamometer chair (Biodex System 4 PRO™, Biodex Medical Systems, Shirley, NY, USA) with the hip flexed to 95° and the knee of the tested leg (PD: self-identified more affected side, controls: self-identified dominant leg) extended to ~160° (180° being full extension) with the contralateral leg on a foot rest. The foot of the tested ankle was secured to the dynamometer footplate with inelastic straps and positioned at the standing ankle angle of the participant measured at the medial malleolus as the angle between the tibia and first metatarsal (PD: 94 ± 8°; Control: 99 ± 4°), aligning the lateral malleolus with the center of rotation of the dynamometer (Figure 1). The standing ankle angle was chosen for the joint position as it best represents the behavior of the muscle and tendon during functional movement of daily life. A 52 cm monitor (1920 × 1200 resolution) was positioned 1 m in front of participants to provide real-time visual feedback of the force signal that was similar across participants and force levels by scaling the feedback relative to the participant’s maximal voluntary contraction (MVC) force. Force was sampled (992 Hz; Power 1401, Cambridge Electronic Design (CED), Cambridge, England), and stored for offline analysis using Spike 2 v7.12 software (CED, Cambridge, England). A B-mode ultrasound probe (ML6-15, 4-15 MHz, GE LOGIQ E9; General Electric, Fairfield, CT, USA) was secured over the distal muscle-tendon junction (MTJ) of the medial gastrocnemius (MG) and Achilles tendon or the muscle belly of the MG using a customized probe holder to record tendon elongation and muscle fascicle shortening, respectively. The ultrasound frame rate was maintained at 31 Hz with an image depth of 2.8 cm. A hyperechoic marker visible on the ultrasound image was secured between the probe and the skin.

Participants performed three isometric plantar flexion MVCs with 2–3 min rest between each contraction to prevent fatigue. From the highest MVC, submaximal force levels of 5, 10, 25, 50, and 75% MVC were calculated for isometric tracking tasks to assess FS. Tracking tasks required participants to match a template displayed on the monitor consisting of a 3-s ramp to the target force, a 5-s plateau, and a 3-s de-ramp to baseline. Two blocks of contractions were performed in a randomized order to obtain recordings of the MG MTJ and muscle belly. Within each block of contractions (MTJ or muscle belly), each force level was performed twice in a randomized order within the block. Each contraction was separated by 2-min rest and 5–10 min of rest was given between blocks during which time the ultrasound probe was repositioned over the muscle belly or MTJ. Following the tracking tasks, participants performed a plantar flexion MVC that was within 5% of the initial MVC to ensure fatigue did not influence the experimental procedures.

### 2.4. Anatomical Measures

**Figure 2:**
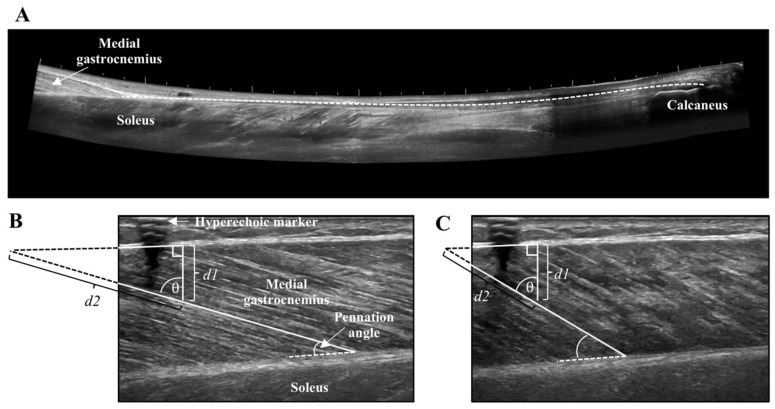
(**A**) LOGIQView^®^ scan of the Achilles tendon showing the length measurement from the calcaneus to the muscle-tendon junction of the medial gastrocnemius. (**B**) Resting and (**C**) contracted medial gastrocnemius fascicle length measurement with extrapolation. Extrapolated portions (d2) were estimated as *d*1/cosθ.

Lever arm length of the foot was measured from the medial malleolus to the head of the first metatarsal. The distance between the medial malleolus and Achilles tendon was the moment arm. Achilles tendon cross-sectional area (CSA) was recorded at the thinnest portion of the tendon using a single static image. Achilles tendon length was captured from the calcaneus insertion to the MTJ of the MG using a LOGIQView^®^ (GE LOGIQ E9) scan (Figure 2A), which is a reliable technique for measuring Achilles tendon length [27]. Combined muscle CSA of the MG and lateral gastrocnemius (LG) was obtained at the thickest portion of the muscles using a LOGIQView^®^ scan.

### 2.5. MVC and Force Steadiness

Torque signals recorded from the Biodex were converted from Newton-meters (Nm) to Newtons (N) of force using the lever arm length of the footplate. Newtons were used in all subsequent analyses as the calculations of tendon mechanics require the use of force in Newtons. Force was analysed offline using custom scripts in Spike 2 v7.12 (CED, Cambridge, England). The highest force recorded of the three MVCs was used for further analyses. Submaximal FS was measured during the middle 3–4 s of the 5-s plateau as the coefficient of variation (CV) of force ((SD of the force signal/mean force produced) × 100) for each contraction within the MTJ block, and the mean of these two contractions is reported. Analyses of CV of force and calculation of tendon mechanics were performed for the MTJ trials and the mean from these two trials is reported. The MTJ trials were chosen for analysis of force and force steadiness as ultrasound analysis for Achilles tendon mechanics of elongation, strain, stiffness, and Young’s modulus (YM) was performed using the MTJ trials in order to provide an accurate representation of the tendon’s mechanical contributions to FS.

### 2.6. Ultrasound Analysis

Tendon and muscle images were measured with the inherent tools of the ultrasound machine (GE LOGIQ E9). The distance from the edge of the screen to the hyperechoic marker was measured to ensure stable probe positioning across all contractions. The CSA of tendon and muscle were measured by tracing the outer border of the Achilles tendon, and combined MG and LG, respectively. Tendon length was measured from the distal MTJ of the MG to Achilles tendon insertion using an open spline trace that allows for measurement along non-linear paths. Tendon elongation and muscle fascicle shortening were measured at the peak of the MVC and during the steady-state plateau of the submaximal contractions. The length of the tendon from the edge of the ultrasound field of view to the MTJ was measured in the resting and contracted states, and the difference between these two values represented tendon elongation. Muscle fascicle length of the MG was obtained in the resting and contracted states by measuring the distance along the fascicle between the lower and upper aponeurosis of the MG. When the insertion of the fascicle onto the upper aponeurosis was not visible in the ultrasound field of view, linear extrapolation was performed using trigonometry and the law of sines (Figure 2B,C) [28,29]. Pennation angle was the angle of fascicle insertion at the lower aponeurosis. The average of two measures for the submaximal contractions are reported for tendon elongation, muscle fascicle shortening, and pennation angle.

### 2.7. Tendon Mechanics

Tendon force was calculated as the quotient of muscle moment and moment arm, with the muscle moment obtained as the product of force and lever arm length. Tendon strain was calculated as the percentage of tendon elongation relative to its resting length, while tendon stress was calculated as the quotient of tendon force and resting tendon CSA. Tendon stiffness was obtained from the slope of the linear portion of the force-elongation relationship from 50 to 100% MVC, and YM was obtained from the slope of linear portion of the stress-strain relationship from 50 to 100% MVC.

### 2.8. Statistical Analysis

Statistical analysis was performed using Statistical Package for Social Sciences version 23 (IBM, Amrok, NY, USA). Resting measures of muscle CSA along with tendon CSA, length, stiffness, and YM were compared between groups using independent samples T-tests. Force steadiness (CV of force) was compared between groups using a 2 (group: PD and control) × 5 (force level: 5, 10, 25, 50, and 75% MVC) repeated measures analysis of variance (ANOVA), while tendon mechanics of elongation, strain and stress were compared using a 2 (group: PD and control) × 6 (force level: 5, 10, 25, 50, 75, and 100% MVC) repeated measures ANOVA. When interactions were present, post-hoc tests between groups were conducted at each force level using independent samples T-tests with Bonferroni corrections. To determine the influence of strength and tendon mechanics on CV of force, MVC, tendon strain, stress, and stiffness were entered into the forward multiple regressions as independent variables with CV of force as the dependent variable. Pearson correlations and forward multiple linear regressions were conducted at low (5 and 10% MVC) and high (25, 50 and 75% MVC) force levels. Separate regressions were conducted as prior studies from our group [16,17] have shown that the contribution of tendon mechanics to CV of force differ between low (2.5–10% MVC) and high (20–80% MVC) force levels. The strength of the correlations were considered as very weak (0–0.3), weak (0.3–0.5), moderate (0.5–0.7), and strong (0.7–1) using the Pearson’s r coefficient. Effect sizes were calculated as eta squared (η^2^) for one-way ANOVAs and partial eta squared (η_p_^2^) for repeated measures ANOVAs, with 0.01, 0.06, and 0.14 representing small, medium, and large effect sizes, respectively. The alpha level was set at 0.05 and values are reported as mean ± SD in text and tables and mean ± SE in figures.

## 3. Results

Persons with PD and controls did not differ in age (*p* = 0.97), height (*p* = 0.52), or weight (*p* = 0.87) (Table 1). Plantar flexion MVC was greater in controls than persons with PD (F = 5.2, η^2^ = 0.25, *p* = 0.037). Resting Achilles tendon length (*p* = 0.778) and CSA (*p* = 0.719), along with muscle CSA of the MG and LG (*p* = 0.174) and standing ankle joint angle (*p* = 0.126) did not differ between groups. Fascicle length (*p* = 0.652) and pennation angle (*p* = 0.813) at rest did not differ between persons with PD and controls (Table 2).

**Figure 3:**
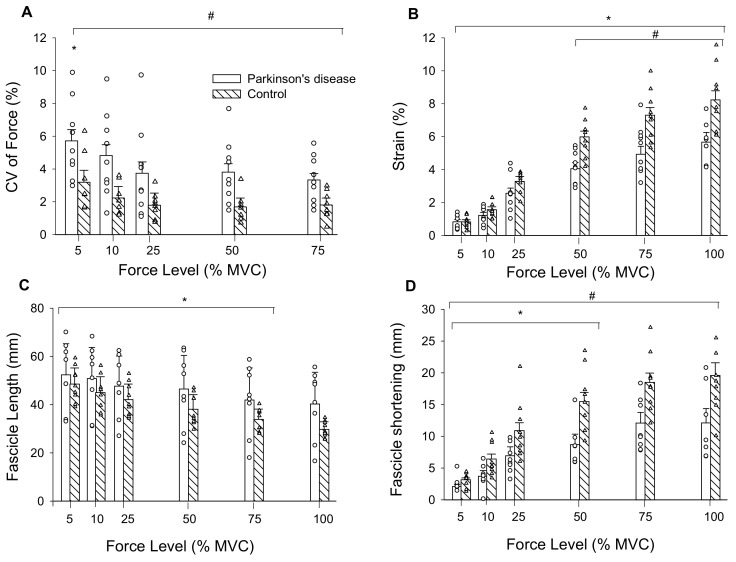
(**A**) CV of force was greatest at 5% MVC compared to all other force levels, and PD were less steady than controls across all force levels as indicated by their higher CV of force; (**B**) tendon strain increased across force levels for both groups and was greater in controls than persons with PD from 50% MVC to MVC; (**C**) Medial gastrocnemius muscle fascicle lengths decreased from 5% MVC to 75% MVC in persons with PD and controls; and (**D**) fascicle shortening increased from 5% MVC to 50% MVC. CV, coefficient of variation; mm, millimeters; MVC, maximal voluntary contraction. #, PD differs from controls; *, differs from all other force levels (*p* < 0.05); Values are mean ± SE.

There were main effects of force (F = 5.52, η_p_^2^ = 0.65, *p* < 0.009) and group (F = 8.76, η_p_^2^ = 0.37, *p* = 0.01) for CV of force. CV of force decreased by 38% from 5 to 25% MVC for both groups and overall, was 67% greater in persons with PD than controls (Figure 3A).

### 3.1. Tendon Mechanics

There was a force by group interaction for tendon elongation (F = 9.49, η_p_^2^ = 0.39, *p* = 0.001). Elongation increased from 5 (1.74 ± 0.72 mm) to 100% MVC (14.41 ± 4.38 mm; force main effect: F = 188.63, η_p_^2^ = 0.93, *p* < 0.001) and the interaction occurred from elongation being greater in controls (12.86 ± 2.89 mm) than persons with PD (8.64 ± 1.93 mm) from 25 to 100% MVC. There was a main effect of force for tendon stress (F = 79.88, η_p_^2^ = 0.83, *p* < 0.001) as it increased from 5 (1.02 ± 0.45 MPa) to 100% MVC (20.2 ± 9.3 MPa). There was no group main effect (F = 0.04, η_p_^2^ = 0.003, *p* = 0.844) or interaction (F = 0.03, η_p_^2^ = 0.002, *p* = 0.871) as stress was similar between persons with PD (8.45 ± 5.18 MPa) and controls (8.83 ± 2.32 MPa). Tendon stiffness (F = 0.036, η^2^ = 0.003, *p* = 0.852) and Young’s modulus (F = 0.058, η^2^ = 0.004, *p* = 0.813) did not differ between persons with PD (86.4 ± 48.8 N/mm; 4.61 ± 2.80 GPa) and controls (82.6 ± 26.5 N/mm; 4.35 ± 0.98 GPa). There was an interaction for tendon strain (F = 9.47, η_p_^2^ = 0.605, *p* = 0.043). Strain increased from 5 to 100% MVC (force main effect: F = 199.64, η_p_^2^ = 0.93, *p* < 0.001) and was greater in controls compared to persons with PD from 50 to 100% MVC (Figure 3B). Because absolute strength potentially influences elongation and strain, and persons with PD were weaker, we set a priori post-hoc to compare these variables between groups at similar absolute forces. At 75% MVC for controls (313.5 ± 78.8 N) and 100% MVC for persons with PD (318.9 ± 99.4 N) elongation (*p* = 0.014) and strain (*p* = 0.030) were greater in controls (15.13 ± 3.30 mm, 7.3 ± 1.5%) compared to persons with PD (11.4 ± 1.98 mm, 5.7 ± 1.2%).

### 3.2. Muscle Fascicles

There was a force by group interaction for fascicle length during submaximal contractions (F = 3.02, η_p_^2^ = 0.18, *p* = 0.05). Fascicle lengths decreased from 5 (46.8 ± 10.5 mm) to 75% MVC (33.6 ± 10.4 mm; F = 65.6, η_p_^2^ = 0.82, *p* < 0.001), and 75% MVC did not differ from 100% MVC (33.5 ± 11.4 mm). Fascicle lengths did not differ between persons with PD (43.4 ± 14.5 mm) and controls (36.3 ± 5.3 mm; F = 1.98, η_p_^2^ = 0.12, *p* = 0.181) across submaximal contraction intensities (Figure 3C).

**Figure 4:**
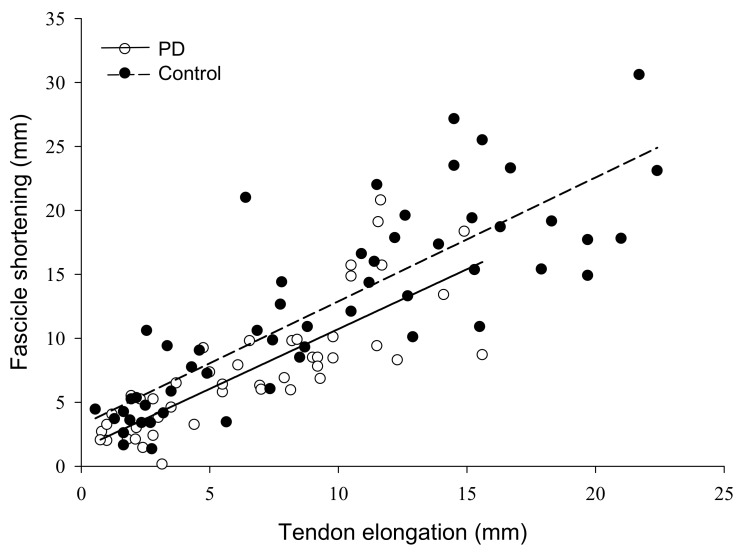
Relationship of muscle fascicle shortening to tendon elongation in persons with Parkinson’s disease and controls. The amount of muscle fascicle shortening was a significant predictor of tendon elongation in both groups, explaining 43% of the variance in tendon elongation for persons with PD, and 60% of the variance in controls (*p* < 0.001). PD, Parkinson’s disease; mm, millimeters.

The force by group interaction for fascicle shortening was non-significant (F = 2.61, η_p_^2^ = 0.16, *p* = 0.09). Fascicle shortening during contraction had a main effect of force (F = 46.47, η_p_^2^ = 0.77, *p* < 0.001) such that fascicle shortening became greater from 5 (2.7 ± 0.98 mm) to 100% MVC (16.3 ± 6.9 mm). There was also a group main effect (F = 11.8, η_p_^2^ = 0.46, *p* = 0.004) as fascicles shortened more in controls (12.3 ± 4.0 mm) than persons with PD (7.6 ± 3.0 mm) (Figure 3D). To control for the influence of absolute strength on fascicle shortening, we compared shortening at similar absolute force levels between groups. At 75% MVC for controls (313.5 ± 78.9 N) and 100% MVC for persons with PD (318.9 ± 99.4 N), fascicle shortening was greater in controls (18.5 ± 4.6 mm) than persons with PD (12.1 ± 5.8 mm) (F = 6.06, η^2^ = 0.30, *p* = 0.027). Pennation angle had a main effect of force (F = 28.52, η_p_^2^ = 0.67 *p* < 0.001) as it increased from 5 (24.3 ± 6.4°) to 100% MVC (36.2 ± 12.4°), but there was no group main effect (F = 1.57, η_p_^2^ = 0.10, *p* = 0.230) or interaction (F = 1.85, η_p_^2^ = 0.12, *p* = 0.186). To determine the contribution of fascicle shortening to tendon elongation, we conducted linear regressions. Fascicle shortening explained 43% of the variance in tendon elongation for persons with PD and 60% of the variance in elongation for controls (*p* < 0.001) (Figure 4).

### 3.3. Correlation and Multiple Regression Analysis

From Pearson’s correlations at low forces, MVC (r = −0.79, *p* < 0.001), strain (r = −0.63, *p* = 0.002) and stress (r = −0.57, *p* = 0.007) were moderately to strongly correlated with CV of force in persons with PD. In age- and sex-matched controls MVC (r = −0.64, *p* = 0.004) and stress (r = −0.43, *p* = 0.049) showed weak to moderate correlations with CV of force (Table 3). At high forces, MVC (r = −0.59, *p* = 0.001), strain (r = −0.45, *p* = 0.014) and stress (r = −0.43, *p* = 0.017) showed weak to moderate correlations with CV of force in persons with PD, and in controls MVC (r = −0.66, *p* = 0.001) was moderately correlated with CV of force and stiffness approached a statistically significant weak correlation (r = −0.35, *p* = 0.06) (Table 3). The multiple regressions at low forces showed that MVC and stress were significant predictors of reduced CV of force in persons with PD (F = 19.73, η_p_^2^ = 0.72, *p* < 0.001), and together accounted for 69% of the variance in CV of force. MVC force was a significant predictor of reduced CV of force in controls (F = 9.74, η_p_^2^ = 0.41, *p* = 0.008), and accounted for 37% of the variance in CV of force (Table 3). At high forces, MVC amplitude was a significant predictor of reduced CV of force in persons with PD (F = 11.79, η_p_^2^ = 0.35, *p* = 0.002), and accounted for 32% of the variance in CV of force, while MVC force and stiffness were significant predictors of reduced CV of force in controls (F = 11.93, η_p_^2^ = 0.57, *p* = 0.001), and together accounted for 52% of the variance in CV of force (Table 3).

## 4. Discussion

Persons with PD were weaker and less steady than age and sex-matched non-PD controls. There was lower tendon strain, tendon elongation, and fascicle shortening in persons with PD than controls in the gastrocnemius–Achilles tendon unit. Further, reduced fascicle shortening capacity for persons with PD culminated in less tendon elongation than controls, in part likely owing to muscle weakness. The lower level of gastrocnemius–Achilles tendon stress accounted for 11% of reduced plantar flexion FS in persons with PD and absolute strength accounted for greater than 35% of the variance in CV of force. Muscle weakness is detrimental to force control directly through absolute force generation as well as indirectly through alterations in tendon mechanics in persons with PD.

Reduced MG fascicle shortening in persons with PD compared to controls could have contributed to lower maximum force-producing capacity as the fascicles likely did not shorten enough to reach optimal length for maximal force production [30]. The reduction in muscle fascicle shortening in persons with PD would reduce the efficiency with which the muscle-tendon unit functions to control and generate force [31]. With tendon stiffness not differing between groups, and fascicle shortening being less in persons with PD, the optimal interaction between the contractile and elastic elements of the muscle-tendon unit were altered. This may impair rapid responses of the muscle and tendon required to maintain standing balance in response to a perturbation, leading to elevated postural instability in persons with PD and greater risk of falls [1]. When matched for absolute force produced at 75% MVC for controls and 100% MVC for persons with PD, fascicle shortening was also less in persons with PD, indicating that absolute force is not the singular contributor to reduced fascicle shortening compared to controls. The non-significant 5° difference in standing ankle angle between groups is unlikely to contribute to differences in fascicle shortening in persons with PD. Prior work from our group has shown that a 10° change in ankle angle does not significantly alter MG fascicle length at rest and during MVC [28], and in this study the fascicle length at rest and across a range of submaximal forces did not differ between groups. It is likely that reduced fascicle shortening in persons with PD arose from decreased capacity to voluntarily activate the muscle [26,32,33,34], as lower voluntary activation of the plantar flexors is associated with reduced MG fascicle shortening in healthy controls [30].

In the current study the PD group were high functioning, as indicated by the average score of 12.7 ± 6.7 on the motor examination portion of the UPDRS [35]. From the multiple regression analyses, higher strength was the greatest predictor of lower CV of force at low and high contraction intensities across groups (Table 2). Muscle strength, combined with MG fascicle shortening explained 43% of the variance in tendon elongation in persons with PD and 60% in controls, and this highlights the importance of understanding factors contributing to maximal force generation in persons with PD. Maximal strength is a factor in FS [14,16,17], and decreased force variability is associated with enhanced force control [2,6,7]. Beyond the importance of this direct contribution of strength, findings of the current study also indicate that as gastrocnemius–Achilles tendon stress increases, force is more concentrated throughout the tendon and this dampens the inherent force variability that arises from neural and muscular elements [13] of FS. Higher tendon stress predicting reduced CV of force was previously reported for the distal biceps brachii tendon in young and old males [16,17]. However, because tendon stress did not differ between persons with PD and controls in our study, it is unlikely to be a factor contributing to neural degenerative disease specific differences; rather a general benefit of increased strength on force control.

An additional factor contributing to reduced MVC and force control in persons with PD compared to controls might also arise from sub-optimal neural drive, as evidence in amplified alpha-band coherence [36], irregular and intermittent [37,38] as well as reduced maximal motor unit discharge rates [39] and voluntary activation [26,32,33,34]. These neuromuscular factors can limit force production capacity and control. Thereby, the optimization of muscle activation would likely mitigate the limiting factor of reduced fascicle shortening and improve muscle-tendon-unit interaction for functional control in persons with PD. It is necessary to consider that our observations are in a group of highly active participants, and are in agreement with a previous report [8] of reduced plantar flexor FS and muscle strength in persons with PD in the ON phase of the medication cycle. Testing during the ON phase of the Levodopa medication cycle was undertaken in the present study and others [8,9] as it provides a measure of task performance and physiological differences in persons with PD at optimal levels of function. We cannot ignore the potential influence of Levodopa on the current findings, and thus results are limited to understanding maximal force production and FS during the ON phase of the medication cycle. Future studies should evaluate ON and OFF phases of the medication cycle to better understand the role of the medication as well as the independent effect of PD.

There are some limitations that must be considered when interpreting the data of the present study. The sample size of nine participants per group is low, particularly in the context of the multiple regression analyses, and therefore these results should be interpreted with caution. The large effect sizes for all comparisons demonstrates that the number of participants was sufficient for the analyses conducted in the present study; however, future studies should confirm these observations as well as investigate this model of the contribution of tendon mechanics to FS in persons with PD using a larger sample size as well as functional measures of daily activity. Persons with PD in the current study were high functioning as indicated by their low scores on the MDS-UPDRS and were tested during the ON phase of their medication cycle. Thus, these findings may not extend to persons with more advanced stages of PD or individuals tested during the OFF phase of the medication cycle. As reported by the persons with PD, we tested the plantar flexors of the more affected limb. However, it is unknown if the observed alterations to muscle-tendon mechanics are restricted to the more-affected side or occur bilaterally as a result of systemic alterations to muscles and tendons arising from disease or drug-therapy. Future studies should evaluate the influence of the medication cycle and side to side difference in muscle-tendon mechanics for persons with PD.

## 5. Conclusions

Our findings indicate that there is an uncoupling of optimal muscle-tendon interaction in persons with PD and that this lessens FS. Although there is similar tendon stiffness in comparison with control participants, persons with PD have less muscle fascicle shortening and thereby less tendon elongation, contributing to the disease specific impairment of FS. This uncoupling of the muscle-tendon unit likely arises from neural factors associated with reduced maximal strength culminating in producing less-steady contractions than age- and sex-matched non-PD controls. The extent to which these changes in the muscle-tendon unit are resultant to the disease or drug therapies remains to be established; however, it is clear that reductions in maximal force generating capacity are influencing the muscle-tendon unit interaction and FS. It is imperative that a greater understanding of maximal muscle activation be achieved in order to enhance force control by optimizing muscle fascicle behaviour and tendon interaction in PD.

## Figures and Tables

**Table 1:** Participant demographics.

	Controls (*n* = 9, 6 Females)	PD (*n* = 9, 6 Females)	95% CI of Difference	
	*Mean* ± SD	*Mean* ± SD	Lower	Upper
Age (years)	70 ± 7	70 ± 5	−6.48	6.25
Height (cm)	163.3 ± 7.1	166.0 ± 9.8	−6.52	6.29
Body Mass (kg)	66.8 ± 14.6	65.8 ± 10.8	−11.21	5.88
MDS-UPDRS Motor Score	N/A	12.7 ± 6.7		
Duration of PD (years)	N/A	6 ± 3		

MDS-UPDRS, Movement Disorders Society Unified Parkinson’s Disease Rating Scale; CI, confidence interval.

**Table 2:** Anatomical measures.

	Controls (*n* = 9, 6 Females)	PD (*n* = 9, 6 Females)	95% CI of Difference	
	*Mean* ± SD	*Mean* ± SD	Lower	Upper
Standing ankle angle (°)	99 ± 4	94 ± 8	−1.75	11.53
MVC (N)	429.2 ± 105.8 *	318.9 ± 99.4	7.70	212.94
Resting tendon length (mm)	207.4 ± 18.3	204.0 ± 30.4	−21.67	28.45
Resting tendon CSA (mm^2^)	38.8 ± 13.5	36.3 ± 14.8	−11.68	16.57
MG and LG Muscle CSA (mm^2^)	1509.7 ± 614.2	1175.3 ± 347.7	−164.37	833.03
Fascicle Length (mm)	48.5 ± 7.1	52.3 ± 13.7	−15.73	8.25
Fascicle Pennation Angle (°)	21.78 ± 5.28	20.81 ± 4.08	−3.96	5.90

PD, Parkinson’s disease; MVC, Maximal voluntary contraction; N, Newtons; CSA, cross-sectional area; MG, Medial gastrocnemius; LG, Lateral gastrocnemius; cm, centimeters; mm, millimeters; CI, confidence intervals. * difference between groups, *p* < 0.05.

**Table 3:** Multiple linear forward regressions of MVC, tendon mechanics and CV of force at low (5, 10% MVC) and high (25, 50, 75% MVC) force levels.

**Low Forces (5, 10% MVC)**					
	**MVC**	**Strain (%)**	**Stress**	**Stiffness**	**MLR**
	**(N)**		**(MPa)**	**(N mm^−1^)**	
PD	−0.787 *	−0.633 *	−0.570 *	n/a	y = 11.805 − 0.017a − 0.802b
	ß = −0.671		*ß* = −0.345		r^2^ = 0.725, adjusted r^2^ = 0.688
Controls	−0.641 *	−0.293	−0.429 *	n/a	y = 8.299 − 0.012a
	ß = −0.641				r^2^ = 0.410, adjusted r^2^ = 0.368
**High Forces (25, 50, 75% MVC)**					
	**MVC**	**Strain (%)**	**Stress**	**Stiffness**	**MLR**
	**(N)**		**(MPa)**	**(N mm^−1^)**	
PD	−0.591 *	−0.446 *	−0.433 *	−0.132	y = 7.829 − 0.013a
	ß = −0.591				r^2^ = 0.349, adjusted r^2^ = 0.319
Controls	−0.655 *	0.067	−0.007	−0.351 ^#^	y = 5.133 − 0.006a − 0.009c
	ß = −0.669			ß = −0.376	r^2^ = 0.570, adjusted r^2^ = 0.522

Pearson correlations and standardized beta weight coefficients (ß) obtained from multiple forward regression analysis. PD, Parkinson’s disease; MVC, maximal voluntary contraction; N, Newtons; MPa, Megapascals; MLR, Multiple linear regressions. a, MVC; b, stress; c, stiffness. *, *p* < 0.05; #, approaches significance at *p* = 0.06.

## References

[1] Jankovic J. (2008). Parkinson’s disease clinical features and diagnosis. J. Neurol. Neurosurg. Psychiatry.

[2] Almuklass A.M., Price R.C., Gould J.R., Enoka R.M. (2016). Force steadiness as a predictor of time to complete a pegboard test of dexterity in young men and women. J. Appl. Physiol..

[3] Jones G.R., Roland K.P., Neubauer N.A., Jakobi J.M. (2016). Handgrip Strength Related to Long-Term Electromyography. Arch. Phys. Med. Rehabil..

[4] Roland K.P., Cornett K.M.D., Theou O., Jakobi J.M., Jones G.R. (2012). Concurrence of Frailty and Parkinson’s Disease. J. Frailty Aging.

[5] Roland K.P., Jones G.R., Jakobi J.M. (2014). Daily electromyography in females with Parkinson’s disease: A potential indicator of frailty. Arch. Gerontol. Geriatr..

[6] Seynnes O., Hue O.A., Garrandes F., Colson S.S., Bernard P.L., Legros P., Fiatarone Singh M.A. (2005). Force steadiness in the lower extremities as an independent predictor of functional performance in older women. J. Aging Phys. Act..

[7] Oshita K., Yano S. (2010). Relationship between Force Fluctuation in the Plantar Flexor and Sustainable Time for Single-leg Standing. J. Physiol. Anthropol..

[8] Skinner J.W., Christou E.A., Hass C.J. (2019). Lower Extremity Muscle Strength and Force Variability in Persons With Parkinson Disease. J. Neurol. Phys. Ther..

[9] Rose M.H., Løkkegaard A., Sonne-Holm S., Jensen B.R. (2013). Tremor irregularity, torque steadiness and rate of force development in Parkinson’s disease. Motor. Control..

[10] Brown P., Corcos D.M., Rothwell J.C. (1997). Does parkinsonian action tremor contribute to muscle weakness in Parkinson’s disease?. Brain.

[11] Ko N.H., Laine C.M., Fisher B.E., Valero-Cuevas F.J. (2015). Force variability during dexterous manipulation in individuals with mild to moderate Parkinson’s disease. Front. Aging Neurosci..

[12] Enoka R.M., Christou E.A., Hunter S.K., Kornatz K.W., Semmler J.G., Taylor A.M., Tracy B.L. (2003). Mechanisms that contribute to differences in motor performance between young and old adults. J. Electromyogr. Kinesiol..

[13] Farina D., Negro F. (2015). Common synaptic input to motor neurons, motor unit synchronization, and force control. Exerc. Sport Sci. Rev..

[14] Jakobi J.M., Haynes E.M.K., Smart R.R. (2018). Is there sufficient evidence to explain the cause of sexually dimorphic behaviour in force steadiness?. Appl. Physiol. Nutr. Metab..

[15] Johannsson J., Jakobi J., Duchateau J., Baudry S. (2015). Do mechanical properties of Achilles tendon influence torque steadiness?. Comput. Methods Biomech. Biomed. Eng..

[16] Smart R.R., Baudry S., Fedorov A., Kuzyk S.L., Jakobi J.M. (2018). Influence of biceps brachii tendon mechanical properties on elbow flexor force steadiness in young and old males. Scand. J. Med. Sci. Sport.

[17] Smart R.R., Kohn S., Richardson C.M., Jakobi J.M. (2018). Influence of forearm orientation on biceps brachii tendon mechanics and elbow flexor force steadiness. J. Biomech..

[18] Feeney D.F., Mani D., Enoka R.M. (2018). Variability in common synaptic input to motor neurons modulates both force steadiness and pegboard time in young and older adults. J. Physiol..

[19] Onambélé G.L., Narici M.V., Maganaris C.N. (2006). Calf Muscle-Tendon Properties and Postural Balance in Old Age. J. Appl. Physiol..

[20] Marusiak J., Jaskólska A., Budrewicz S., Koszewicz M., Jaskólski A. (2011). Increased Muscle Belly and Tendon Stiffness in Patients with Parkinson’s Disease, as Measured by Myotonometry. Mov. Disord..

[21] Koh S., Roh J., Kim J., Oh K., Kim B., Kim G., Park B., Kim S., Yoon J. (2008). Ultrasonographic findings of shoulder disorders in patients with Parkinson’s disease. Mov. Disord..

[22] Tan B., Double K.L., Burne J., Diong J. (2016). Tension-referenced measures of gastrocnemius slack length and stiffness in Parkinson’s disease. Mov. Disord..

[23] Orselli M.I.V., Franz J.R., Thelen D.G. (2017). The effects of Achilles tendon compliance on triceps surae mechanics and energetics in walking. J. Biomech..

[24] Csapo R., Maganaris C.N., Seynnes O.R., Narici M.V. (2010). On muscle, tendon and high heels. J. Exp. Biol..

[25] Bohm S., Mersmann F., Arampatzis A. (2015). Human tendon adaptation in response to mechanical loading: A systematic review and meta-analysis of exercise intervention studies on healthy adults. Sport Med. Open.

[26] Stevens-Lapsley J., Kluger B.M., Schenkman M. (2012). Quadriceps muscle weakness, activation deficits, and fatigue with parkinson Disease. Neurorehabil. Neural Repair.

[27] Ryan E.D., Rosenberg J.G., Scharville M.J., Sobolewski E.J., Thompson B.J., King G.E. (2013). Test-Retest Reliability and the Minimal Detectable Change for Achilles Tendon Length: A Panoramic Ultrasound Assessment. Ultrasound Med. Biol..

[28] Kuzyk S.L., Smart R.R., Simpson C.L., Fedorov A., Jakobi J.M. (2018). Influence of fascicle length on twitch potentiation of the medial gastrocnemius across three ankle angles. Eur. J. Appl. Physiol..

[29] Simpson C.L., Kim B.D.H., Bourcet M.R., Jones G.R., Jakobi J.M. (2017). Stretch training induces unequal adaptation in muscle fascicles and thickness in medial and lateral gastrocnemii. Scand. J. Med. Sci. Sport.

[30] Arampatzis A., Mademli L., De Monte G., Walsh M. (2007). Changes in fascicle length from rest to maximal voluntary contraction affect the assessment of voluntary activation. J. Biomech..

[31] Lichtwark G.A., Wilson A.M. (2008). Optimal muscle fascicle length and tendon stiffness for maximising gastrocnemius efficiency during human walking and running. J. Theor. Biol..

[32] Stelmach G., Tesdale N., Phillips J., Worringham C. (1989). Force production characteristics in Parkinson’s diease. Exp. Brain Res..

[33] Hammond K.G., Pfeiffer R.F., LeDoux M.S., Schilling B.K. (2017). Neuromuscular rate of force development deficit in Parkinson disease. Clin. Biomech..

[34] Folland J.P., Haas B., Castle P. (2011). Strength and activation of the knee musculature in Parkinson’s disease: Effect of medication. NeuroRehabilitation.

[35] Shulman L.M., Gruber-Baldini A.L., Anderson K.E., Fishman P.S., Reich S.G., Weiner W.J. (2010). The clinically important difference on the unified parkinson’s disease rating scale. Arch. Neurol..

[36] Laine C.M., Valero-Cuevas F.J. (2020). Parkinson’s Disease Exhibits Amplified Intermuscular Coherence During Dynamic Voluntary Action. Front. Neurol..

[37] Milner-Brown H., Fisher M., Weiner W.J. (1979). Electrical Properties of Motor Units in Parkinsonism and a Possible Relationship With Bradykinesia. J. Neurol. Neurosurg. Psychiatry.

[38] Glendinning D., Enoka R.M. (1994). Motor Unit Behavior in Parkinson’s Disease. Phys. Ther..

[39] Agapaki O., Christakos C., Anastasopoulos D. (2018). Characteristics of rest and postural tremors in Parkinson’s disease: An analysis of motor unit firing synchrony and patterns. Front. Hum. Neurosci..

